# Functional Condition of Patients after Unilateral Hip Arthroscopy in the Process of FAI—Femoroacetabular Impingement: A Case–Control Study and Preliminary Report

**DOI:** 10.3390/jcm10051023

**Published:** 2021-03-02

**Authors:** Olga Nieszporska, Aleksandra Truszczyńska-Baszak

**Affiliations:** Faculty of Rehabilitation, Józef Piłsudski University of Physical Education, 01-968 Warsaw, Poland; o.nieszporska@gmail.com

**Keywords:** femoroacetabular impingement, range of motion, strength, hip joint, quality of life, functional condition, arthroscopic surgery

## Abstract

Introduction: Femoroacetabular impingement is a commonly recognized condition among people with hip pain. Aim: The aim of this study was to assess how arthroscopy and physiotherapy treatment influenced the quality of life and functional condition of patients after arthroscopic femoroacetabular impingement (FAI) surgery. Materials and methods: We examined 19 people for the study and included 12 (6 men and 6 women). Their mean age was 40.1 ± 9.7 years. Manual and digital goniometers were used for the range of motion (ROM) measurements, and a dynamometer for muscle strength was used. Results from the operated limb were compared to the nonoperated healthy limb. We examined the patient’s health and well-being using the Harris Hip Score (HHS) and Short-Form Health Survey (SF-36) scales. The mean follow-up period was 21.2 months. Results: The postsurgery mean range of motion for all movements was lower in the operated limb. Statistically significant differences between limbs in ROM were observed for flexion, abduction, extension, and external rotation. Muscle strength was comparable between hip joints, except extension and adduction, which were statistically significantly weaker. The mean strength of the hip flexors and internal rotators was higher in the operated limb. After surgery, 67% of patients returned to exercise at the same or higher level. The mean HHS results were good, with values of 88.00 ± 11.48. The SF-36 scores were >50. Conclusion: After surgery and physiotherapy of FAI, ROM remained lower in the operated limb. Flexion and rotations remained to cause pain. The strength of flexors and internal rotators improved, and there was a high rate of return to sport.

## 1. Introduction

Femoroacetabular impingement (FAI) results from abnormalities in contact between the femoral head and neck and the acetabulum. The surfaces of the joints, the shapes of which are abnormal, lose their physiological adjustment, and repeated impingement may lead to damage of the cartilage and the acetabular labrum, consequently leading to early degenerative changes. Ganz et al. recognized three different FAI types: (1) cam type—femoral head and neck deformity, (2) pincer type—acetabular deformity, and (3) mixed type [1,2,3].

Impingement is accompanied by pain in the area of the greater trochanter or the groin. In a study by Larson et al., as many as 96.6% of patients with hip pain were diagnosed with impingement [4]. The causes of FAI have not yet been thoroughly studied. It is believed to be common in young adults with an active lifestyle. High-intensity sports, particularly in adolescence, may predispose one toward impingement [5,6]. Some other predisposing factors include a frequent extreme repeated range of movements or childhood diseases such as epiphyseal or developmental dysplasia, slipped capital femoral epiphysis, and Legg–Calve–Perthes disease [3,5,7]. However, other predisposing factors include increased alpha and Wiberg angles. Literature reviews inform on the impact of certain genetic factors that may influence the development of degenerative disease [6,8]. FAI is usually believed to be a primary disease, and it may develop as a result of the following: prolonged epiphysis, ossification resulting from congenital abnormalities, infections and autoimmunological reactions, inflammatory diseases, and injuries [3,7]. The development of impingement may be impacted by: extensive femoral antetorsion, acetabular protrusion or retroversion, hip varus or valgus, and femoral fractures [9,10]. The surgical treatment of osteotomy and an overdeveloped greater trochanter are believed to cause secondary FAI [3,5].

The aim of this study was to determine the extent to which arthroscopy and physiotherapy influence the function of operated hips and, consequently, patients’ quality of life.

## 2. Materials and Methods

The main premise was to conduct a comparative analysis of the functional states of the operated and healthy limb. To conduct the analysis, we took measurements of ranges of motion and muscle strength. The measurement positions were chosen to enable: (1) the patient to take the starting position; (2) the patient to perform the movement; (3) the researcher to stabilize the patient’s position; (4) the researcher to read the measurement; (5) the swift conduct of the measurement. Satisfaction with the surgical interventions and whether the interventions resulted in patients’ increased quality of life were evaluated with questionnaires. Additionally, we studied patients’ well-being and general health.

This study evaluated the functional state of adult patients after FAI arthroscopy. The study population was homogeneous in terms of diagnosis (*n* = 12 with mixed-type FAI), surgery (the same experienced orthopedic surgeon and procedure—during standard surgery in all patients, all of the components of FAI such as the labral tear, damaged articular cartilage, and bony changes were treated with the assistance of the arthroscope), physiotherapy treatment (the same procedure), and time passed since surgery (minimum of 6 months; mean: 21.2 months). After the surgery, each patient had undergone the same standard physiotherapy, which lasted 4–6 months.

The exclusion criteria were other surgical interventions to any of the lower limbs, as well as the qualification of the so-far nonoperated limb for future surgery.

**Figure 1:**
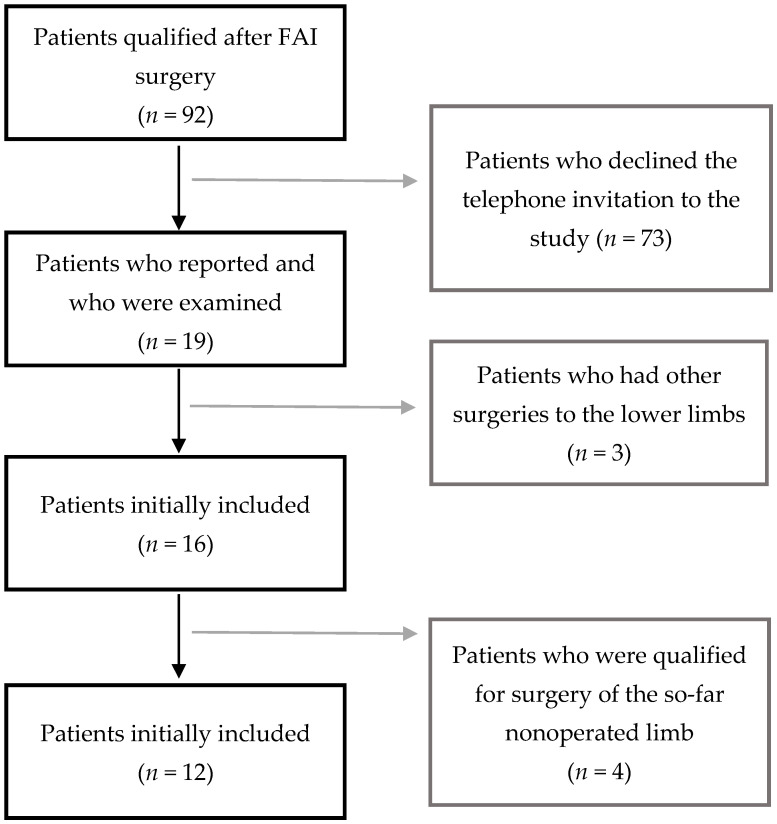
Study inclusion process.

Ninety-two patients after hip arthroscopy qualified for the study. All the surgeries were conducted in the period from 2015 to 2019. The patients were invited to participate in the study by phone. Nineteen patients reported to the clinic, and all of them were examined. Twelve patients met the inclusion criteria (Figure 1)—6 men and 6 women. Their mean age was 40.1 ± 9.7 years. The time that had passed since the surgery was no shorter than 6 months—a mean of 21.2 ± 12.9 months. This time span allowed qualifying the results as short-term. The right limb was operated on in 5 patients, and the left limb was operated on in the remaining 7 patients.

The mean time prior to the surgery, when the pain or limitations to the range of motion appeared that inhibited the patients in their daily living activities, was 26.89 ± 21.22 months. Three patients reported that the pain did not inhibit their daily life activities, whereas all the patients reported pain during physical activity. The mean time between the first symptoms and the surgery was 25.50 ± 19.52 months (ranging from 4 to 60).

Postsurgery recommendations included a painless flexion of range 0–90° with the use of a continuous passive motion (Continuous Passive Motion—no specific manufacturer) device for 6 to 8 h daily for 6 weeks. The device was not used if patients had a chondrofiller. Before surgery, patients did not receive standardized physiotherapy. Each patient received standard instructions and was trained on how to walk with crutches while in hospital after the surgery.

All patients reported to the clinic between 2 and 4 weeks after surgery. Depending on the patient’s condition, range of motion limitations, and functional and sport-related needs, physiotherapy lasted 4–6 months. In the early stage of therapy, patients’ exercises aimed at improving blood circulation, traction, and stretching. Lymphatic drainage, kinesio taping, and cryotherapy were used 3 times a day for 2 weeks to reduce swelling. Cryotherapy was also used later if there was swelling. Additionally, patients received a magnetic field and electrostimulation of the quadriceps femoris muscle for 2 months. Crutches were phased out gradually for movement in a painless range after a period of a total ban on limb loading defined by the surgeon. Moderate load was introduced 4 to 8 weeks postsurgery and was gradually increased until Weeks 7 and 8. If the cartilage was operated upon, this period was prolonged by a mean of 2 weeks. Patients were recommended not to walk without crutches for longer than 2 h per day until 3 months postsurgery. Further exercise was chosen individually and depended to a large extent on the patients’ functional abilities and age, as well as the size of the injury.

We obtained consent from the Senate Ethics Committee No. 01-14/2020. Patients were informed about the detailed procedures and the aim of the study, and they signed an informed consent form to participate in the study prior to participation in the study procedures. All patients could ask necessary questions or withdraw from the study. Patients were instructed to report any pain they experienced to the person conducting the study procedures.

The procedures started with a homogeneous warm-up on a stationary Kettler bike. Patients cycled for a period of 5 min with constant resistance from the pedals (women—5, men—7) and a cadence from 60 to 70.

Passive movement ranges were then measured for both hips. Flexion, extension, and both rotations were measured with a digital goniometer, and abduction and adduction were measured with a manual goniometer.

**Figure 2:**
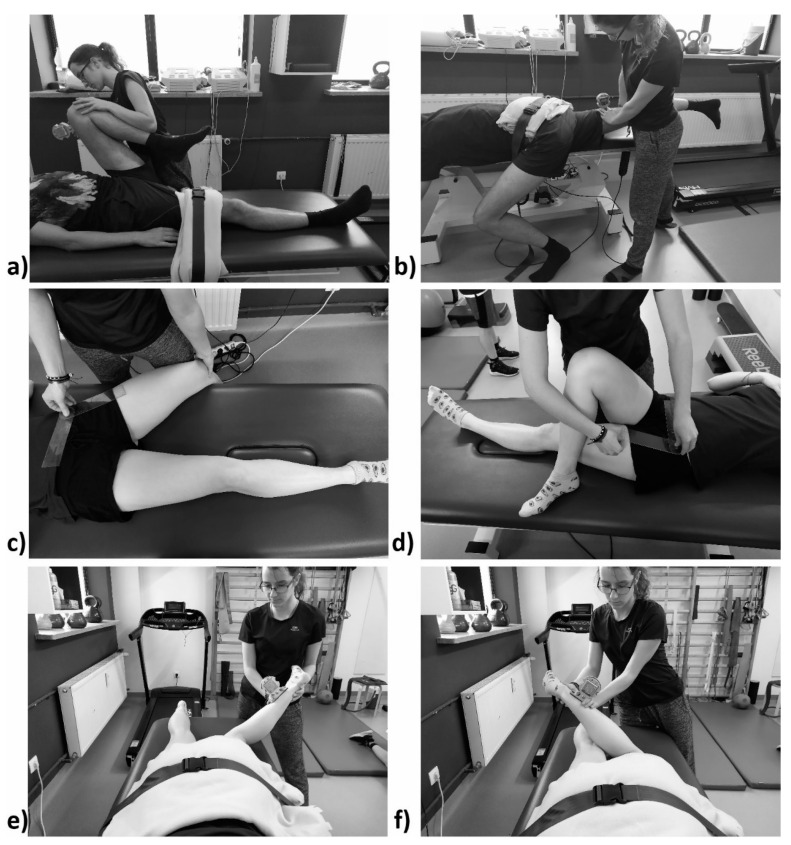
Measurement positions for range of motion: (**a**) flexion; (**b**) extension; (**c**) abduction; (**d**) adduction; (**e**) internal rotation; (**f**) external rotation. Authors’ own materials.

The flexion measurement was conducted in the following manner: the hip and knee were flexed with the patient lying on their back. The inclinometer was placed just over the patella, and it was reset in a neutral position. Stabilization was achieved with a belt placed above the knee. The extension measurement was conducted with the patient lying on their front. The inclinometer was placed above the knee and reset in a neutral position. Stabilization was achieved with a belt placed on the level of the sacral bone, with the opposite limb lowered off the couch so that the extension movement was not transferred to the lumbar region of the spine. External rotation measurements were conducted with the patient lying on their front, with the knees bent at a 90° angle, stabilized with a belt over the pelvis. The inclinometer was reset at the wall and was placed over the medial malleolus for internal rotation and over the lateral malleolus for external rotation. For the abduction and adduction movements, we used the traditional recommendations by Zembaty on the measurement position and on the placement of the goniometer [11]. The end of the range of motion was subjectively registered at the moment when the spina iliaca anterior superior moved, on the opposite side for abduction and on the same side for adduction. Figure 2 presents the measurement positions.

**Figure 3:**
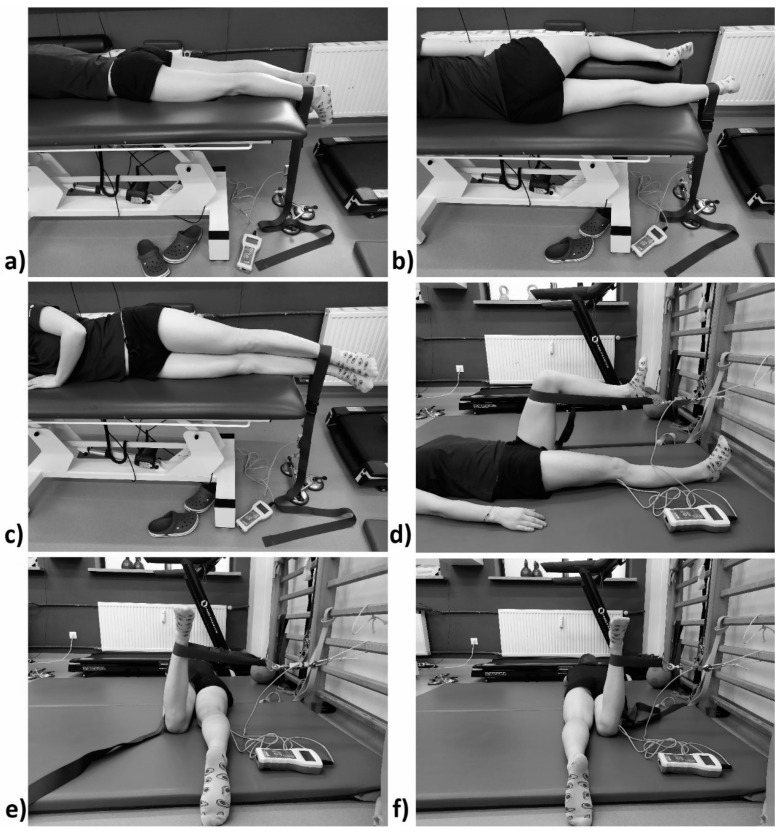
Positions for strength measurements: (**a**) flexion; (**b**) extension; (**c**) abduction; (**d**) adduction; (**e**) internal rotation; (**f**) external rotation. Authors’ own materials.

We then measured the strength of each movement with a digital dynamometer (N) (Figure 3). Patients were asked to perform a maximum isometric tension lasting 3 s three times. The highest measurement was then chosen for analysis. Before performing the movement, the patient was informed on how to perform the tension correctly. Each time, the device was fixed at a straight angle to the limb axis with a belt, and on the other side, it was fixed to a sucker or to wall bars. The dynamometer was reset before fixing, with the tension equaling zero. Figure 3 shows the muscle strength measuring positions.

To finish the tests, the patients were asked to complete 2 questionnaires on their own, apart from the Harris Hip Score (HHS) questionnaire, where the last 3 questions (on deformities, movement scales, total result) were completed by the researcher.

1. The Short-Form Health Survey (SF-36) is used for the subjective assessment of patients’ quality of life. It comprises 36 statements. They are divided into 8 categories: physical functioning (PF), physical role functioning (RP), bodily pain (BP), general health perceptions (GH), emotional role functioning (RE), social role functioning (SF), mental health (MH), and vitality (VT). Question 2 on health transition (HT) within the past year was assessed separately. The categories were grouped into two components: physical component (PCS) and mental (MCS) mental component. The highest score for a question denoted the highest quality of life. The scores for each category were then calculated into points on a 0–100-point scale. The norm for the population was set at 50 points [12,13].
2. The Harris Hip Score (HHS) is used to assess the functional state of the hip. It assesses the following: pain in the operated joints, range of motion, ability to move, ability to sit, and ability to put on shoes and socks. Each answer is adequately scored. The higher the score, the better the functional state. The maximum score is 100 points [14]. For the purposes of this study, results <70 were considered poor, results between 70 and 79 were considered fair, results between 80 and 89 were considered good, and results between 90 and 100 were considered excellent [15].
3. We created a questionnaire on health and well-being for the purposes of this study. It supplements information that previous questionnaires lack. It contains inter alia questions on pain before and after surgery that is felt during daily life activities and during sports activities. Additionally, the subjects provided information on the frequency of sports activity, discomfort in the nonoperated limb, subjective improvement, and other orthopedic surgeries. All the questions were asked personally by the first author. Doubts or misunderstandings did not arise during the interview.

## 3. Results

The mean range of motion in the nonoperated limb was always greater than in the operated limb. We observed a statistically significant difference (*p* < 0.05) in the following movements: flexion, abduction, extension, and external rotation (Table 1).

The accuracy of the goniometer was 1°. The measurement error of the researcher was 0.98°. The results correlated with another observer. The rank agreement of the measurements was full, i.e., the order of measurements in both evaluators was the same. The W-Kendall judges’ concordance factor was calculated to be 1, and Cronbach alpha was 0.98.

Muscle strength measurements were compared between the limbs. There was a statistically significant difference in extension and adduction (Table 2).

### 3.1. Questionnaire Results

Mean pain reduction was 6.33 ± 2.53 on the visual analogue scale (VAS) scale (0–10). Before the surgery, the subjects exercised, with a mean frequency of 3.65 ± 1.86 times/week, and after surgery, it was 3.72 ± 1.89 times/week. Most of the subjects (*n* = 5) felt slight discomfort during physical activity both before and after surgery, and three subjects did not perform any sport because of the pain and did not perform any sport after the surgery. One surveyed subject declared they had not performed physical activity before and after the surgery. Another subject stopped exercising after the surgery, even though before they exercised regularly, approximately three times per week.

### 3.2. HHS Results

The mean total HHS score was 88.00 ± 11.48 points (ranging from 63 to 97), which denoted a good result, and eight subjects (67%) had excellent scores. Two subjects (17%) had poor scores. The range of motion in all subjects was in the highest or highest but one range, with mean values of 210.42 ± 28.76° (ranging from 171 to 279). Six patients (50%) reported limping.

### 3.3. Quality of Life SF-36 Results

**Figure 4:**
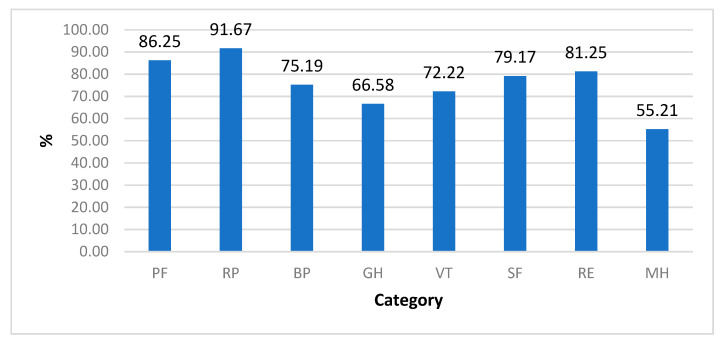
Mean results in 8 categories, SF-36 in %. PF—physical functioning; RP—physical role functioning; BP—bodily pain; GH—general health perceptions; RE—emotional role functioning; SF—social role functioning; MH—mental health; VT—vitality.

The mean results for all eight categories were higher than 50. Therefore, these results were higher than in the general population (Figure 4). Patients assessed their physical health (a mean of 80.00 points ± 13.71) better than their mental health (a mean of 68.92 ± 18.66).

## 4. Discussion

The difficulty in this study was enrolling a suitably large study population. Some subjects who qualified for the study were reluctant to report to the clinic or lived far away from the city.

Arthroscopic surgery has been gaining popularity lately, and it is the most common method chosen for the surgical treatment of femoroacetabular impingement [16]. It has comparable effectiveness to the open method. Short- and medium-term results suggest a reduced risk of complications [17]. Medium- and long-term results prove a statistically significant difference in the quality of life [18]. Other authors have noted that arthroscopy increased the chances of returning to sports, improved the HHS score, and shortened physiotherapy. This type of treatment had better effects in comparison with conservative treatment too [16,19].

Kekatpure et al. assessed the effectiveness of conservative treatment. They compared a group of operated subjects to subjects without surgery. The results were verified after three months, and then at a two-year follow-up. In the early phase, there was no difference between the groups. The authors noted that it was usually younger subjects who had undergone surgical treatment. It was concluded that surgeries should only be performed when pain persists, when patients express their willingness to undergo surgery, or when there are visible changes in the imaging tests [20]. In the analyzed subjects, there was no attempt at conservative treatment, or physiotherapy was ineffective and did not reduce discomfort, so all patients decided to undergo surgery. Bennell et al. compared physiotherapy conducted by a specialist with their own program. It was found that individually designed programs controlled by physiotherapists may improve results from the patients’ perspective and shorten the regeneration process [21]. According to Spencer-Gardner et al., an appropriate postsurgical physiotherapeutic process should involve exercise progression and load training adjusted to the surgical technique used. After a four- or five-phase process, patients have good clinical and functional results, and most of them assess their state as normal or almost normal [22].

Our study found that arthroscopic surgery and postsurgical physiotherapy seem to provide effective treatment for femoroacetabular impingement. Öhlin et al. conducted a five-year follow-up assessment of treatment results with several scales and had similar results, where 84.6% of their subjects were satisfied with the surgery [23].

In our study, the total HHS score was good (88.00), which is comparable to results from the study by Spencer-Gardner et al. (80.1) [22]. Beck et al. concluded that a higher modifies HHS score and a lower VAS score were found in the following type of patient: nonsmoker, male, with a smaller alpha angle. Additionally, the following factors were important in determining whether the patient had a satisfying treatment result: patient acceptable symptom state (PASS), sporty lifestyle, low body mass index (BMI), small alpha angle, lack of cartilage damage, and lack of chondromolation [24].

According to Kahlenberg et al., there is a moderate relationship between a patient’s satisfaction and postoperative modified HHS score. Therefore, satisfaction assessment should be used as a complementary tool in functional assessment [25]. Most patients after arthroscopy who were surveyed with HHS were satisfied with the effects and had PASS and a minimal clinically important difference (MCID) [26].

Physical activity is another factor that shows the effectiveness of treatment. Patients who perform sports both recreationally and professionally had reduced pain and increased functionality after arthroscopic surgery [27]. In their literature review, Memon et al. outlined the high frequency of returning to sports activities after arthroscopic surgery. In total, 93% of patients returned to sports. The best results were achieved in children, professional athletes, and patients with short-lasting symptoms prior to the surgery. Pain reduction and improvement of function were found in a majority of subjects [28]. Frank et al. analyzed returns to recreational cycling (70% outside, 30% inside). Almost 100% of subjects returned to sports approximately 4–5 months postsurgery, most of them with results better than or comparable to these from before the surgery [29]. In our study, eight patients (67%) declared that the frequency of their physical activity during the week was on the same level or higher in comparison to their activity before surgery. The same number of patients declared that they did not have discomforts after surgery, that these discomforts did not disturb them in performing sports activities, or that they were able to perform sports with discomfort.

Studies that present measurements of muscle strength and range of motion often compare postsurgery patients with clinical controls [30,31,32]. Most of the available literature provides an account of patients’ states before and after surgical intervention [31,33]. This type of assessment was impossible to conduct within our study, as patients reported to the physiotherapeutic clinic only after their surgery. There were two studies published in 2019, which, apart from the above, also presented a comparison of the operated limb and the nonoperated limb, similar to this study. In the study by Freke et al., measurements were taken before the surgery, as well as three and six months after the surgery. In the operated limb, three months postsurgery, there was an improvement in the range of motion of flexion. In internal and external rotation, the range of motion remained on a constant level, lower than the nonoperated limb. The differences in muscle strength between the limbs were small. Flexion recovered the longest, whereas extension quickly regained the level from before the surgery [31]. Wörner et al. compared postsurgery patients to healthy controls as well as the operated limb to the nonoperated limb 6–10 months postsurgery. In addition, they compared return to sports, and they conducted tests on jumping ability, balance, and dexterity. They found that hip flexion strength and passive range of motion of flexion remained reduced. There was a significant difference in the flexion ROM between the operated limb and nonoperated limbs [32].

In our study, there were statistically significant differences in the flexion, abduction, extension, internal rotation range of motion, and extension and adduction muscle strength. It is difficult to relate our results to the greater population because of the small size of the study population. We have found, however, that the mean range of motion in the nonoperated limb had higher values than in the operated limb. The study by Tijsen et al. had a similar scheme to our study. The measurements of internal rotations found a significant difference between the limbs. They also found a slight difference in the adduction and abduction movements [34].

Value of the study. The significant value of the study was the homogeneity of the study population. All the surgeries were conducted by the same orthopedic surgeon, the postsurgical physiotherapy was conducted in the same clinic, and the measurements were taken by the same researcher. All the patients had the same and specific diagnosis: FAI. The results presented are complex as they include dynamometer measurements of muscle strength and range of motion. They assess the functional state, and they also involve the psychological state and the subjective improvement assessment by the patients.

Limitations of the study. The fundamental and the most important limitation of the study was the small size of the study population. As a result, it was difficult to draw significant conclusions and relate the results to the general population. We compared the operated limb to the nonoperated limb, which may be at risk of developing FAI in the future. There was no comparison of the results with the clinical control group or the patients’ states prior to the surgery, which may make a reliable assessment of treatment difficult. The study population was not homogeneous in terms of age or physical activity.

It is necessary to conduct follow-up studies in the future in order to understand the issue better. It is necessary to involve a larger study population and compare the results from before the surgery with those after surgery. Moreover, it may be essential to conduct long-term studies that would allow for determining the length of the benefits of the treatment, as well as the risk of repeated surgery and developing discomforts in the so-far nonoperated limb.

## 5. Conclusions

After the FAI surgery, range of motion remained lower in the operated limb compared to the nonoperated limb. The muscle strength of both limbs was comparable. Total HHS scores and SF-36 quality of life were very good.

We found a moderate relationship between the functional state of the patients, HHS score, and assessment of the quality of life.

## Figures and Tables

**Table 1:** Range of motion in both limbs in degrees (°).

Movement	Operated Limb	Nonoperated Limb	Difference	*p*
Flexion	117.00 (±12.05)	126.42 (±9.11)	9.42	0.02
Abduction	17.50 (±4.68)	21.17 (±6.31)	3.67	0.01
Adduction	9.83 (±4.11)	10.17 (±2.62)	0.33	0.65
Extension	19.83 (±7.22)	24.58 (±9.54)	4.75	0.01
Internal rotation	26.50 (±17.55)	32.17 (±15.16)	5.67	0.10
External rotation	39.58 (±8.49)	48.33 (±9.55)	8.75	0.04

**Table 2:** Muscle strength in both limbs in N.

Movement	Operated Limb	Nonoperated Limb	Difference	*p*
Extension	185.58 (±85.92)	210.43 (±88.28)	24.85	0.002
Abduction	165.40 (±65.88)	171.77 (±59.49)	6.37	0.40
Adduction	160.12 (±71.68)	180.95 (±61.98)	20.83	0.02
Flexion	223.82 (±69.21)	218.67 (±69.41)	−5.15	0.43
Internal rotation	109.80 (±45.00)	105.50 (±39.27)	−4.30	0.43
External rotation	121.67 (±52.97)	133.27 (±56.52)	11.60	0.10

## Data Availability

The data presented in this study are available on request from the corresponding author.
